# Mixed messages: wild female bonobos show high variability in the timing of ovulation in relation to sexual swelling patterns

**DOI:** 10.1186/s12862-016-0691-3

**Published:** 2016-06-30

**Authors:** Pamela Heidi Douglas, Gottfried Hohmann, Róisín Murtagh, Robyn Thiessen-Bock, Tobias Deschner

**Affiliations:** Department of Primatology, Max Planck Institute for Evolutionary Anthropology, Deutscher Platz 6, D-04103 Leipzig, Germany; Faculty of Biology, Ludwig-Maximilians-University of Munich, Biocenter Großhaderner Str. 2, D-82152 Planegg-Martinsried, Germany

**Keywords:** Primate, Sexual signalling, Fecundity, Endocrine analysis, LC–MS/MS, Estrogen, *Pan paniscus*, Pregnanediol, Mate guarding

## Abstract

**Background:**

The evolution of primate sexual swellings and their influence on mating strategies have captivated the interest of biologists for over a century. Across the primate order, variability in the timing of ovulation with respect to females’ sexual swelling patterns differs greatly. Since sexual swellings typically function as signals of female fecundity, the temporal relation between ovulation and sexual swellings can impact the ability of males to pinpoint ovulation and thereby affect male mating strategies. Here, we used endocrine parameters to detect ovulation and examined the temporal relation between the maximum swelling phase (MSP) and ovulation in wild female bonobos (*Pan paniscus*). Data were collected at the Luikotale field site, Democratic Republic of Congo, spanning 36 months. Observational data from 13 females were used to characterise female swelling cycles (*N* = 70). Furthermore, we measured urinary oestrone and pregnanediol using liquid chromatography–tandem mass spectrometry, and used pregnanediol to determine the timing of ovulation in 34 cycles (*N* = 9 females).

**Results:**

We found that the duration of females’ MSP was highly variable, ranging from 1 to 31 days. Timing of ovulation varied considerably in relation to the onset of the MSP, resulting in a very low day-specific probability of ovulation and fecundity across female cycles. Ovulation occurred during the MSP in only 52.9 % of the analysed swelling cycles, and females showed regular sexual swelling patterns in *N* = 8 swelling cycles where ovulation did not occur. These findings reveal that sexual swellings of bonobos are less reliable indicators of ovulation compared to other species of primates.

**Conclusions:**

Female bonobos show unusual variability in the duration of the MSP and in the timing of ovulation relative to the sexual swelling signal. These data are important for understanding the evolution of sexual signals, how they influence male and female mating strategies, and how decoupling visual signals of fecundity from the periovulatory period may affect intersexual conflict. By prolonging the period during which males would need to mate guard females to ascertain paternity, the temporal variability of this signal may constrain mate-guarding efforts by male bonobos.

**Electronic supplementary material:**

The online version of this article (doi:10.1186/s12862-016-0691-3) contains supplementary material, which is available to authorized users.

## Background

In social animals, interactions between males and females are often driven by reproductive interests [1–4]. In species where males compete for access to females, male behaviour may be influenced by female secondary sexual characteristics (e.g., [5–7]). Males who are sensitive to female sexual signals often change their behaviour in ways that maximise their mating opportunities and reproductive success [8]. Females may incur high costs from displaying sexual signals if the signals incite aggressive or coercive behaviour from males [9]. Per contra, females may derive benefits from sexual signals if they buffer aggression from males [10], facilitate recruiting agonistic support in conflicts, enhance access to food sources [11], elicit parental care [12], or minimise the risk of infanticide by enabling females to mate polyandrously [13]. Depending on the mating strategies of males, females may exploit sexual signals in order to maximise the benefits that they derive [14]. Females may do so either by displaying prolonged sexual signals which temporally exceed the phase of elevated fecundity [15, 16], or by obscuring cyclic changes in fecundity [17]. Empirical evidence suggests that female sexual signals offer a particularly effective strategy to manipulate the behaviour of males [18–20].

One type of sexual signal, which is absent in most taxa but common in nonhuman primates, is female sexual skin swellings (hereafter sexual swellings). In primate species that possess this morphological trait, the skin surrounding the female genitalia changes in size, shape, turgidity, and colour during the follicular phase, and usually culminates in maximum size and turgidity around the periovulatory period [21, 22]. Among the catarrhine primates, some species have particularly conspicuous or exaggerated sexual swellings, e.g., chimpanzees [16, 23], macaques [24], baboons [25], and bonobos [26]. The majority of species that possess exaggerated sexual swellings live in multimale-multifemale groups [27]. This lends support to the theory that these morphological signals play a role in intersexual communication and function to influence mating patterns.

Several hypotheses pertaining to the evolution and function of exaggerated sexual swellings have been reviewed in the primate literature [28–30] with the breadth of hypotheses reflecting the variance in the reliability of this trait. The reliable indicator hypothesis [31] proposes that sexual swellings are honest signals of female quality or condition. For example, sexual swellings seem to reflect aspects of long-term reproductive value in female chacma baboons [32]. The graded-signal hypothesis [28] proposes that variation in the size of exaggerated sexual swellings conveys information about the probability of ovulation, allowing males to adjust their mating strategies according to their individual monopolisation potential. Across primate species, the precision with which sexual swellings signal ovulation varies considerably along a continuum. Near one end of the continuum, sexual swellings are relatively precise indicators of ovulation through which females signal their reproductive status and fecundity to conspecifics (e.g., [33–36]). Thereby, females attract mating partners and potentially incite male-male competition [36]. Further along the continuum, sexual swellings indicate ovulation with less precision and reliability [37], although the highest probability of ovulation still occurs when swellings are at their peak size during the maximum swelling phase (MSP) [16, 38, 39]. In these species, males often adjust their mate-guarding efforts in accordance with the signal, and females are able to bias or confuse paternity depending on the amount of variability in the timing of ovulation and in the duration of the MSP. The more a female’s MSP duration exceeds the period of fecundity and the greater the variability in the timing of ovulation within the MSP, the less precisely swellings indicate the timing of ovulation. In turn, it becomes increasingly difficult for males to time their mating efforts accurately within the period of high fecundity [30].

Early invasive studies of captive nonhuman primates revealed that cycles of sexual swelling are modulated by hormonal events of the menstrual cycle, with tumescence of the sexual swelling caused by an increase in oestrogens, while progesterone can act synergistically with oestrogens or inhibit their effect and thereby result in swelling detumescence [23, 40–43]. However, a female’s social environment also can affect ovarian function and swelling size [44–46]. Flexibility in the expression of sexual swellings has been noted in studies where different social cues or events caused females to display situation-dependent swellings [47, 48] or deceptive swellings in the absence of ovulation [49]. Variability in the flexibility and accuracy of this signal therefore merits further investigation.

The same hormones underlying female sexual swellings, i.e., oestrogens and progesterone, are also used in many studies of human and nonhuman primates to determine the occurrence of ovulation and to pinpoint its timing [16, 50]. One species that is notable in terms of variability in timing of ovulation in relation to sexual swelling patterns and female reproductive strategies is the bonobo (*Pan paniscus*). To date, only a few studies have measured ovarian hormones in female bonobos, and all were conducted exclusively in captivity [51–54]. These studies indicated that the variability in the timing of ovulation in relation to sexual swelling patterns in bonobos is considerably higher than in other species, with ovulation sometimes occurring outside the MSP [53]. However, because energy intake can alter hormone levels and reproductive functioning, and is generally higher and more regular in captive settings, field studies are critical for furthering our understanding of the reproductive endocrinology and sexual signalling of wild bonobos.

Bonobos live in multimale-multifemale societies in which females exhibit extended sexual receptivity and mating is polygynous and polyandrous. During the ovarian cycle, female bonobos show conspicuous changes in the size, shape, and firmness of their sexual swellings and the skin surrounding their perinea [55, 56]. Bonobos are a particularly interesting species in which to study signal accuracy and honesty in tandem with reproductive endocrinology, since females’ sexual swellings often display an unusually lengthy MSP both in captivity [57, 58] and in the wild [59]. Furthermore, wild female bonobos have a shorter period of lactational amenorrhoea compared to chimpanzees [60–62] and often resume having sexual swelling cycles within 1–2 years following parturition. Finally, bonobos are an interesting species in which to study sexual signalling given that females are often dominant over males [63], males do not engage in sexual coercion, and mating behaviour is used in a variety of contexts [64, 65].

In this study, we investigated the relationship between ovarian hormones and sexual swellings in wild female bonobos. Our specific objectives were to: (1) quantify the duration of sexual swelling cycles of wild female bonobos; (2) assess the intraindividual and interindividual variability in the duration of the MSP; (3) quantify reproductive hormone levels by measuring urinary oestrone and pregnanediol to generate hormone profiles across swelling cycles; (4) examine the temporal relation between sexual swellings and ovulation; and (5) investigate factors which may influence the duration of the MSP. Our final aim was to assess the predictability of ovulation in wild bonobos by examining how accurately sexual swellings signal ovulation and fecundity in female bonobos.

## Methods

### Study site and subjects

Data were collected from the Bompusa community of wild bonobos at the Luikotale field site, located near the southern sector of Salonga National Park, Democratic Republic of the Congo [66]. Data collection spanned a three-year period, from December 2010 to December 2013. During this study, the Bompusa community consisted of 13–14 mature females (estimated age > 10 yrs; Table 1), 6–7 mature males (estimated age > 10 yrs), and 11–19 dependent offspring. We based age estimates on physical features such as body size, dentition, physical signs of aging, and sexual swellings. Females were considered to be nulliparous if they immigrated to the community as young females, were not observed to have given birth, and if the appearance of their nipples suggested that they had not lactated previously. All bonobos were fully habituated and individually recognisable, and all females were long-term, permanent residents, with the exception of one female (Djulie) who immigrated to the community in 2012. Eight females gave birth during the study period.

**Table 1:** Identity and characteristics of mature females in the Bompusa community

Female	Parity	Dominance rank	Estimated age of female^a^ (years)	Age of youngest dependent offspring^a^ (years)	Date of most recent parturition	Swelling cycles analysed	Hormone cycles analysed
Djulie	N	9	9–11	–	–	–	–
Gwen	P; M	3	15–18	3.3	Sep 2012	4	–
Iris	M	1	> 25	0.8	Mar 2010	6	–
**Luna**	N; P	5	11–13	–	Mar 2013	7	4
**Martha**	M	1	> 30	–	circa 2003	11	4
Nina	N; P	7	11–13	–	Jan 2011	1	–
**Olga**	M	2	> 25	1.6	Oct 2012	6	5
**Paula**	M	1	> 25	0.5	Oct 2012	5	3
Polly	N	8	9–11	–	–	2	–
**Rio**	M	2	> 25	> 5	May 2012	4	3
**Susi**	P	6	15–18	1.4	May 2009	6	4
**Uma**	P; M	3	15–18	2.3	Oct 2012	7	4
**Wilma**	P	4	11–13	–	Feb 2011	5	4
**Zoe**	M	2	> 25	0.5	Jun 2010	6	3
					Total	70	34

^a^At the beginning of the study (December 2010)

Focal females are indicated in bold typeface. Parity abbreviations are as follows: *P* primiparous, *M* multiparous, *N* nulliparous. A dominance rank of 1 indicates the highest rank

### Assessment of sexual swellings and the maximum swelling phase (MSP)

Following a previously established methodology for bonobos [57, 67], sexual swellings of all mature females observed each day were visually inspected and assigned a score (1 to 4) based on relative tumescence, firmness, labial occlusion, and lustre. Swelling stage 4 was termed the maximum swelling phase (MSP), and was characterised by fully taut and swollen tissue of the sexual swelling, a sturdy appearance during locomotion, labial occlusion, and lustre [67].

The duration of the MSP was calculated from the first to the last day of swelling stage 4. In several cycles, female swelling scores temporarily dropped to stage 3 during the MSP. If the swelling returned to stage 4 following the temporary drop to stage 3 and did not detumesce beyond stage 3, then these temporary periods (range: 1–4 days) of swelling stage 3 were included in the overall duration of the MSP. Only cycles that did not have a sample gap greater than one day at the onset or end of the MSP were used to analyse variation in the duration of the MSP. A total of 70 swelling cycles from 13 females fulfilled this criterion (Table 1). When there was a one-day sample gap, we used a more conservative estimate of the MSP and only counted the number of days that we observed a female with swelling stage 4.

### Urine sample collection

Urine sample collection targeted a subset of nine focal females (see Table 1), representing females of different ages and reproductive states, e.g., cycling, pregnant, and lactating. During swelling stages 1–3, samples were collected from focal females at regular intervals, e.g., every two to three days, to monitor hormone levels and to enable detection of any ovulations outside the MSP. We attempted to collect urine samples every day at the onset of stage 4 (the MSP) and for at least five days after the onset of detumescence. Urine was collected from females using a previously described technique [68]. In brief, the underside of large leaves was used to capture urine from arboreal females. If this was not possible, urine was collected by aspiration from ground vegetation using disposable plastic pipettes. Only urine not contaminated with faeces was collected. Urine was pipetted into 2-ml polypropylene tubes and stored in liquid nitrogen within 12 h of collection. Samples were transferred from liquid nitrogen to dry ice for transport to the Max Planck Institute for Evolutionary Anthropology (MPI-EVA) in Leipzig, Germany, where they were stored at –20 °C until analysis.

### Hormone extraction and measurement

We selected a subset of 710 urine samples for analysis in the Endocrine Laboratory of the MPI-EVA. Samples were selected with the objective to equally represent each of the nine focal females with several cycles collected from different points in time. We followed the methods described by Hauser et al. [69] to extract oestrone (E1)–an oestrogen metabolite–and pregnanediol (Pd)–a urinary metabolite of progesterone–amongst other steroid hormones. In brief, 100 μl of each urine sample was spiked pre-extraction with a 50 μl solution containing deuterated internal standards. Progesterone-d9 (Dr. Ehrenstorfer, Augsburg, Germany) and Estrone-d4 (Sigma Chemical Co., St. Louis, MO, USA) were chosen to quantify and correct for matrix loss in Pd and E1 respectively. To de-conjugate the steroid glucuronides, we performed an enzymatic hydrolysis using β-glucuronidase from *E. coli* (Sigma Chemical Co., St. Louis, MO, USA). This was followed by solvolysis to cleave the steroid sulphate conjugates.

We measured levels of urinary E1 and Pd following the liquid chromatography–tandem mass spectrometry (LC-MS/MS) method [69] to generate profiles of hormone levels across female swelling cycles. This was done using a high performance Waters Alliance 2695 (Waters, Milford, MA) for chromatographic separation. Subsequently, mass spectrometry was performed on a Quattro Premier XE (Micromass, Manchester, UK) with a Z-spray ESI interface. For more details see Additional file 1. We measured creatinine in each sample [70, 71] and indexed hormone values with creatinine to control for variations in the volume and concentration of the voided urine. All hormone values are expressed in ng/mg creatinine (Cr).

### Interpretation of hormone profiles and definitions

The timing of ovulation and corresponding onset of the luteal phase was calculated from urinary Pd excretion profiles. A sustained rise in Pd levels above a defined threshold value of two standard deviations above the mean of the preceding three to five baseline values [72] was used to non-invasively detect ovulation, in adherence with other studies [16, 17, 73]. Based on the finding that the Pd rise in urine occurs one to three days after the serum luteinising hormone peak [50, 74] and to account for the time lag in the excretion of hormones into urine, the day preceding the Pd rise was defined as the day of ovulation and designated as day zero. However, there may be an error of one day in this determination.

The fecund phase, or periovulatory period, was defined as the day of ovulation plus the three preceding days [16]. Since sperm remain viable in the female tract for several days, copulations that occur two to three days prior to ovulation can result in conception. This window of fecundity, when conception is most likely to occur, was delineated based on findings from studies of captive nonhuman primates [75] and humans [76]. Cycles were termed anovulatory if a sustained rise in Pd was not detected in the urine. The interovulatory interval (IOI) was calculated from the day of ovulation to the day of ovulation in the next cycle (*N* = 8 IOI). The interswelling interval (ISI) was used to infer duration of swelling cycles, and was calculated from the onset of the MSP to the onset of the MSP in the subsequent cycle (*N* = 37 ISI). We used the IOI and the ISI as proxies for cycle duration. We did not use the intermenstrual interval, because menstruation could not be detected reliably in all females. The duration of the luteal phase was calculated from the day of ovulation to the first day of observed menstruation, in cycles where menstruation was detected. Hormone profiles were plotted against female swelling scores to examine the temporal relation between ovulation and swelling cycles. In cases where ovulation occurred outside of the MSP, we associated ovulation with the MSP that was closest in terms of the number of days.

### Statistical analysis

We estimated the day-specific probability of ovulation by dividing the number of times we observed ovulation on a particular cycle day by the total number of cycles examined. This was calculated in accordance with Deschner et al. [16] using the equation:

$$ P\left(T = t\right)=\frac{n_t}{n},\kern0.75em t = 1,\ 2,\ 3\dots, $$

where *t* represents a specific cycle day (relative to the start of the MSP), *n*_*t*_ is the number of cycles in which ovulation occurred on day *t*, and *n* is the total number of cycles.

Likewise, the day-specific probability of fecundity was estimated following Deschner et al. [16] using the equation:

$$ P\left(X(f)=1\right)={\displaystyle \sum_{t=f}^{f+3}}P\left(\mathrm{T}=\mathrm{t}\right), $$

where *(X(f) = 1)* represents a day on which a female could conceive, and *P(T = t)* is as stated above. The day-specific probability of fecundity, also referred to as the probability of conception [77, 78], is a measure of the probability that copulation could lead to conception on any given day.

### Models and test predictors

We ran six analyses using linear mixed models (LMMs) and Generalised Linear Mixed Models (GLMMs) [79, 80]. All models were fitted in R version 3.2.4 [81] using the functions lmer or glmer of the package lme4 [82]. We assessed collinearity amongst predictors by deriving Variance Inflation Factors (VIFs) [83, 84], using the function “vif” of the package “car” [85] based on standard linear models lacking the random effects. For each model, we first assessed the significance of the fixed effects as a whole [86], by comparing the fit of the full model to a null model using a likelihood ratio test [87]. The null models lacked the fixed effects. We then determined the significance of the individual fixed effects using likelihood ratio tests [88], comparing the full model with reduced models, dropping the fixed effects one at a time. For each model, we obtained model stability by comparing estimates obtained from the full model with estimates from models with the levels of the random effects excluded one at a time. Since the estimates did not vary greatly [89], all model results were robust.

Female dominance rank and social status can influence ovarian hormone levels [90], the duration of the swelling phase [91], and the duration of cycles and interbirth intervals [91, 92]. Therefore, we included female rank as a fixed effect in all models. Social dominance was assessed and ranks were generated (see Table 1) using the ADAGIO method, version 1.1 [93]. Dominance ranks ranged from one (highest rank) to nine (lowest rank). Female ranks were z-transformed to a mean of zero and a standard deviation of one, prior to fitting each model [94].

### MSP duration model

Previous studies of nonhuman primates have proposed that female parity and reproductive state may influence the duration of the MSP (e.g., [53, 95, 96]). Based on these findings, we fitted a LMM to investigate to what extent these factors influenced the duration of female bonobos’ MSPs at Luikotale. As fixed effects, we included female parity as a factor with two levels (“multiparous” and “primiparous”), female reproductive state as a factor with two levels (“cycling”, i.e., experiencing ovulatory cycles, and “not cycling”, e.g., pregnant), number of days since parturition, and female dominance rank as a quantitative predictor. Because the number of days since parturition was skewed and we wanted to avoid outliers that would bias the results, we square root transformed this variable. To control for potential seasonal variation we also included the sine and cosine of the Julian date (after multiplying it by 2 × π and then dividing by 365.25, to convert date into a circular variable). Such a representation of season allowed us to model the response showing a sinusoidal periodicity with a period duration of one year; that is, the response peaking once per year (for more details see [97]). As a random effect, we included female identity (ID). To keep type one error rate at the nominal level of 0.05, random slopes [88, 94] of days since parturition as well as sine and cosine of date within female ID were included in the model. Random slopes of the other fixed effects could not be included, because they varied either rarely within females (e.g., reproductive state) or not at all (female parity).

The sample size for this model was 53 MSPs from 11 females. Since MSP duration was rather skewed, we square root transformed it before fitting the model. This resulted in residuals fulfilling the assumptions of normality and homogeneity (verified by visual inspection of a QQ-plot and residuals plotted against fitted values). Collinearity, assessed by VIFs, appeared to be a minor issue between parity and female rank (maximum VIF: 3.5). Therefore, we fitted two additional LMMs: one excluding the test predictor parity, and a second excluding female rank. These models were fitted and checked in the same way as the main model. Collinearity was not an issue in these additional models (maximum VIF: 1.2).

We tested for absence of influential cases by excluding females one at a time from the data and comparing the estimates derived with those obtained for the full data set, which revealed the model to be stable. To test the overall effect of the fixed effects [86], we compared the full model with a null model that comprised only the effects of season and the random effects, using a likelihood ratio test [87]. Furthermore, to test for significant interindividual variation above and beyond the four fixed effects, we compared the full model to a reduced model lacking only the random intercept term of female ID. The sample size for this reduced model was the same as the full model.

### ISI duration model

We fitted a GLMM with poisson error distribution and log link function to investigate variation in the ISI duration. The sample size for this model was 37 ISIs from 13 females. As fixed effects, we included female parity as a factor with three levels (“multiparous”, “nulliparous”, and “primiparous”), female reproductive state as a factor with two levels (“cycling” and “early lactation”), and female rank as a quantitative predictor. Following O’Malley et al. [98], we defined early lactation as 0–24 months following parturition, based on evidence that lactation, and the energetic burden of lactation, are most intense in chimpanzees during the first 24 months following parturition [99, 100]. We chose not to include days since parturition as a fixed effect so that the nulliparous females could be included in this model. As a random effect, we included female ID. We could not control for seasonal variation or days since parturition in this model because including them resulted in model stability problems and the model being too complex given the sample size [84]. As an overall test of the effect of the three fixed effects, we compared the full model with a null model that lacked the fixed effects and comprised only the random effect of female ID. Collinearity was not an issue (maximum VIF: 2.1). The model was overdispersed (dispersion parameter = 1.74) which makes the model anticonservative. However, given the model results, this did not represent a problem (see Results section).

### Female rank and occurrence of ovulation model

We fitted a GLMM with binomial error structure and a logit link function to investigate the occurrence of ovulation. Specifically, we tested the influence of female rank and reproductive state on whether or not a cycle was ovulatory (yes/no). The sample size for this model was 34 cycles from nine females. As fixed effects, we included female reproductive state as a factor with two levels (“cycling” and “early lactation”) and female rank as a quantitative predictor. As a random effect, we included female ID. Collinearity was not an issue (maximum VIF: 1.0). To determine the significance of the fixed effects, we compared the full model with a null model that lacked the fixed effects and comprised only the random effect of female ID.

### Female rank and timing of ovulation model

We fitted a GLMM with binomial error structure and a logit link function to investigate the timing of ovulation. Specifically, we tested the influence of female rank and reproductive state on whether or not ovulation occurred during the MSP (yes/no). The sample size for this model was 26 cycles from nine females. As fixed effects, we included female reproductive state as a factor with two levels (“cycling” and “early lactation”) and female rank as a quantitative predictor. As a random effect, we included female ID. Collinearity was not an issue (maximum VIF: 1.0). To determine the significance of the fixed effects, we compared the full model with a null model which comprised only the random effect of female ID.

## Results

### Female sexual swelling cycles, interswelling interval (ISI), and interovulatory interval (IOI)

All females had cyclical fluctuations in the relative degree of tumescence, firmness, lustre, and labial occlusion of their sexual swellings. Although there was variation in female swelling characteristics, e.g., the absolute size of sexual swellings, the four different swelling stages could be reliably distinguished within each female. The mean cycle duration, inferred from the ISI, was ($\overline{X}$ ± SD) 41.2 ± 13.8 days (range: 20–74 days; *N* = 37 cycles; *N* = 13 females). Mean cycle duration calculated from the IOI was very similar, ($\overline{X}$ ± SD) 40.8 ± 6.8 days (range: 30–51 days; *N* = 8 cycles; *N* = 6 females). Mean duration of the luteal phase was ($\overline{X}$ ± SD) 9.5 ± 1.2 days (range: 8–11 days; *N* = 6 cycles; *N* = 4 females). Overall, there was no significant effect of female parity, rank, and reproductive state on the ISI duration (full-null model comparison: *χ*^2^ = 0.90, df = 4, *p* = 0.925; see Additional file 2: Table S1).

In four out of six pregnancies, females had sexual swelling cycles including a MSP during at least the first three months of pregnancy (range: 1–6 months; *N* = 6 females). Females resumed showing sexual swelling cycles as early as three months following parturition ($\overline{X}$ ± SD = 7.0 ± 4.8 months; range: 3–14 months post-parturition; *N* = 5 females).

### Duration of the maximum swelling phase (MSP)

**Fig. 1:**
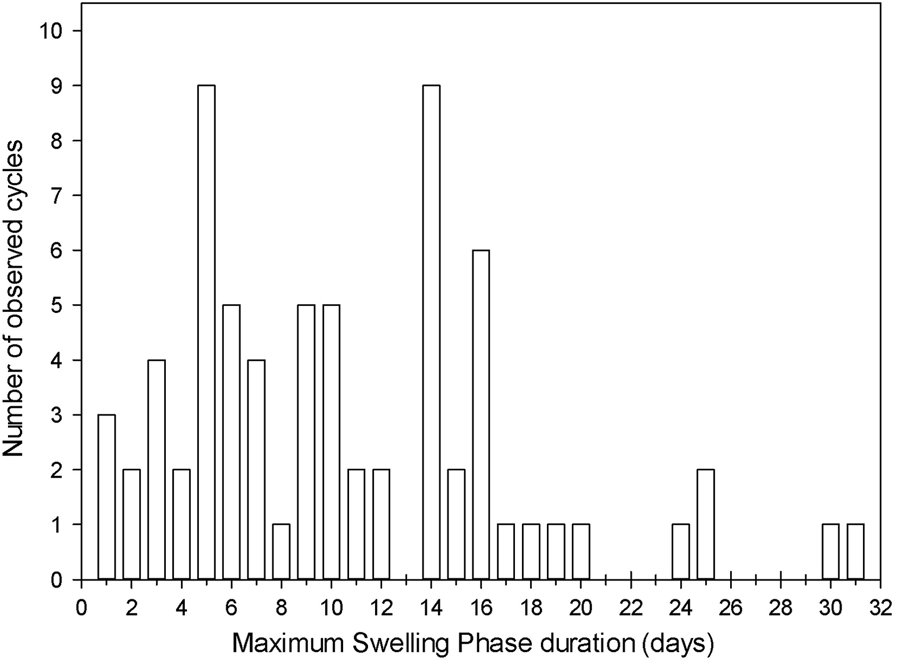
Frequency distribution of the duration of females’ MSPs (*N* = 70 cycles; 13 females)

**Fig. 2:**
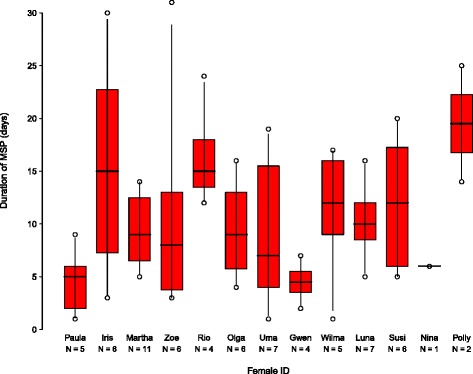
Intra-individual variation in MSP duration. Shown are medians (thick horizontal lines), quartiles (boxes), percentiles (2.5 and 97.5 %, vertical lines), as well as the minimum and maximum of each female’s MSP (circles). Females are arranged from highest-ranking (left) to lowest-ranking (right)

Duration of the MSP ranged from 1–31 days ($\overline{X}$ ± SD = 10.6 ± 6.8 days; *N* = 70 cycles; *N* = 13 females; Fig. 1). MSP duration was highly variable both within and between females (Fig. 2). The full-null model comparison did not reveal a significant effect of female parity, number of days since parturition, female rank, and reproductive state on the MSP duration (*χ*^2^ = 7.01, df = 4, *p* = 0.136; Additional file 2: Table S2). The model excluding parity was also nonsignificant (full-null model comparison: *χ*^2^ = 5.66, df = 3, *p* = 0.129; Additional file 2: Table S3). Only the model which excluded female rank had a marginally nonsignificant effect on the MSP duration (full-null model comparison: *χ*^2^ = 6.36, df = 3, *p* = 0.095; Additional file 2: Table S4). More specifically, we found a tendency for the MSP duration to increase as the number of days since parturition increased. Although this predictor had the strongest effect on the duration of the MPS, it was not statistically significant (see Additional file 2: Table S4). Furthermore, MSP duration did not vary significantly between individuals (*χ*^2^ = < 0.001, df = 1, *p* = 1.00). There was little variation between mean values of MSP duration from different reproductive states (Table 2). The mean MSP duration was shorter for females during early lactation, i.e., within 24 months of parturition, than during other reproductive states. This corroborates the finding of a tendency for MSP duration to increase as days since parturition increases, as suggested by the results of the MSP model that excluded female rank.

**Table 2:** Mean duration of the maximum swelling phase (MSP) for each reproductive state

Reproductive state	Mean duration of the MSP	SD	Number of cycles	Number of females
Cycling	11.8	7.1	41	11
Pregnant	10.5	5.9	4	3
Early lactation	8.6	6.1	25	9

### Hormone profiles

**Fig. 3:**
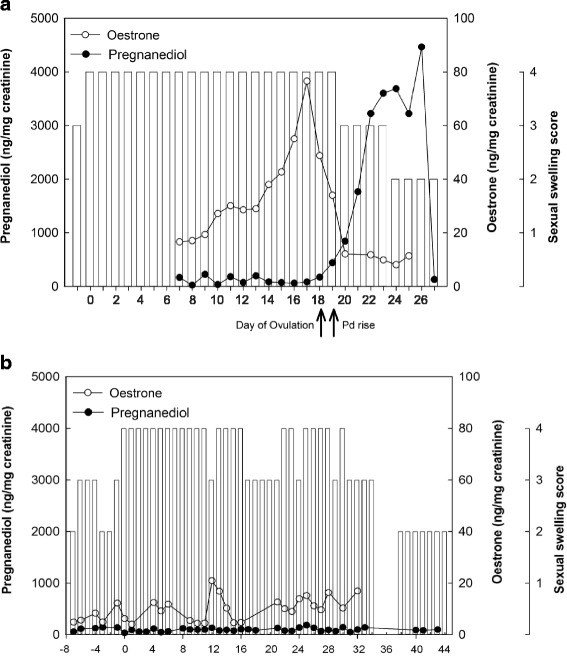
Profiles of urinary oestrone (open circles) and pregnanediol (closed circles) in relation to changes in sexual swelling size (open bars) in two individual cycles of two female bonobos: (**a**) a presumably ovulatory cycle from Susi; and (**b**) an anovulatory swelling cycle from Zoe. The day relative to the first day of the MSP (day 0) is indicated on the x-axis

Representative profiles of urinary E1 and Pd in relation to the pattern of sexual swelling during a nonconception cycle in an individual female are depicted in Fig. 3a. All presumably ovulatory profiles showed a well-defined pattern of Pd excretion, characterised by consistently low levels during the follicular phase followed by markedly elevated levels during the luteal phase. Although profiles of E1 were more variable between and within cycles, levels of E1 gradually increased and reached a discernible peak one to three days before the defined postovulatory Pd rise in 96.2 % of ovulatory cycles.

In contrast to the presumably ovulatory cycles, in eight swelling cycles (collected from *N* = 5 non-pregnant females) there were only small fluctuations in levels of E1, and increases in E1 were not followed by a significant or sustained rise in Pd (Fig. 3b). The corresponding profiles of urinary Pd also showed a very different pattern in these eight cycles compared to the ovulatory cycles, with no significant rise that would be indicative of ovulation and the start of the luteal phase. In some of these cycles, the rise in E1 may be suggestive of follicular development comparable to the follicular phase of an ovulatory cycle. However, given the consistently low levels of Pd in these cycles, it is highly unlikely that ovulation and a subsequent luteal phase occurred. These eight swelling cycles were therefore considered to be anovulatory. Anovulatory cycles occurred at various points in time throughout a female’s interbirth interval, ranging from 10.8 to 42.3 months after parturition (see Additional file 3: Table S8). The full-null model comparison did not reveal a significant effect of female rank and reproductive state on whether a cycle was ovulatory or anovulatory (*χ*^2^ = 0.45, df = 2, *p* = 0.799; Additional file 2: Table S5).

### Composite profiles

**Fig. 4:**
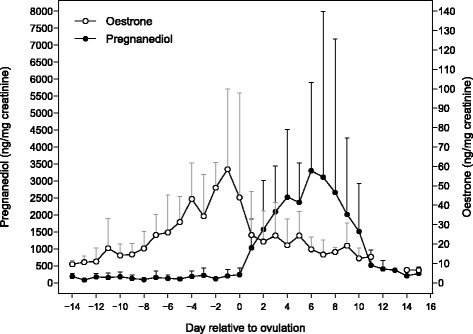
Composite profiles of urinary E1 (open circles) and Pd (closed circles), derived from 26 ovulatory cycles from 9 females. Values are presented as mean ± SD and aligned to the day of ovulation (day 0), one day prior to the significant postovulatory rise in Pd, indicating the onset of the luteal phase

We created composite profiles of urinary E1 and Pd, representing the 26 ovulatory cycles of the nine focal females (Fig. 4). Concentrations of E1 and Pd varied between females and between cycles of individual females, with the largest variation occurring during periods when hormones were at peak values. Mean E1 levels fluctuated between 9.6 ± 2.0 and 17.8 ± 15.4 ng/mg Cr during days –14 to –9 (early follicular phase), then increased to a peak mean level of 63.5 ± 40.1 ng/mg Cr two days before the Pd rise. Mean levels of Pd were consistently low on days during the early follicular phase (range of $\overline{X}$ ± SD: 83.7 ± 38.9 to 182.8 ± 95.3 ng/mg Cr), then increased rapidly to a mean peak concentration of 3303.3 ± 2597.6 ng/mg Cr during the luteal phase.

### Timing of ovulation relative to the maximum swelling phase (MSP)

**Fig. 5:**
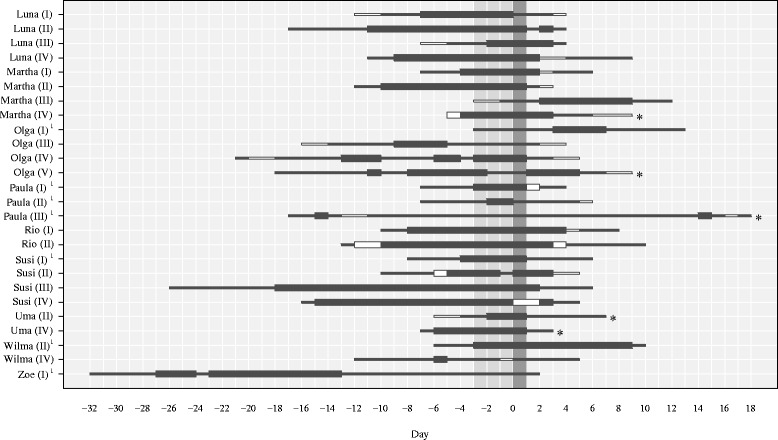
Duration and timing of the MSP relative to the day of ovulation (day 0) in ovulatory cycles. Asterisks indicate conception cycles; the shaded area indicates the fecund phase. Thick black bars indicate the MSP; thin black bars indicate swelling stage 3; white bars indicate sampling gaps. Female names and cycle numbers are shown on the y-axis. ^(1)^Indicates that this cycle occurred during early lactation. All other cycles occurred > 24 months postparturition

**Fig. 6:**
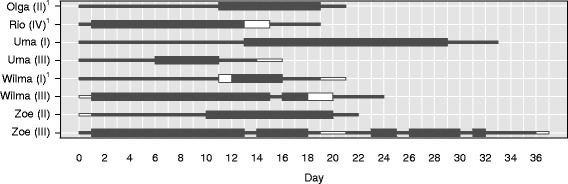
Duration of the MSP in anovulatory swelling cycles. Thick black bars indicate the MSP; thin black bars indicate swelling stage 3; white bars indicate sampling gaps. Female names and cycle numbers are shown on the y-axis. ^(1)^Indicates that this swelling cycle occurred during early lactation. All other swelling cycles occurred > 24 months postparturition

The timing of ovulation relative to the onset and end of the MSP varied considerably, both between females and within females (*N* = 26 ovulatory cycles; see Fig. 5). In 18 cycles (69.2 % of ovulatory cycles), ovulation occurred during the MSP, between 2 and 18 days after the onset of the MSP (which corresponded to 1 to 9 days before the end of the MSP). In the remaining eight ovulatory cycles (30.8 %), ovulation occurred outside of the MSP, either during swelling stage 3 before the onset of the MSP (*N* = 2) or during swelling stage 3 after the onset of detumescence (*N* = 6). Overall, there was no significant effect of female rank and reproductive state on whether or not ovulation occurred during the MSP (full-null model comparison: *χ*^2^ = 4.46, df = 2, *p* = 0.107; Additional file 2: Table S6). The MSP duration in each of the eight anovulatory swelling cycles (Fig. 6) was within the range of the MSP duration of the ovulatory cycles.

### Day-specific probability of ovulation and fecundity

**Fig. 7:**
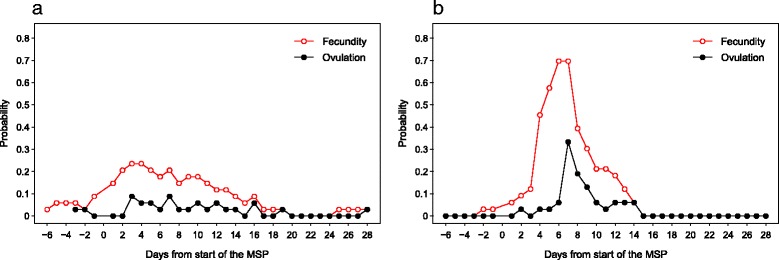
Probability of ovulation (black lines, black circles) and fecundity (red lines, white circles) relative to the first day of the MSP of: (**a**) bonobos (*N* = 34 cycles); and (**b**) chimpanzees at Taï National Park (*N* = 33 cycles) (calculations based on data from [16])

Due to the high variability in the timing of ovulation relative to the MSP, the day-specific probability of ovulation was very low on any given day of a female’s cycle (Fig. 7a). Days three and seven of the MSP revealed the highest probability of ovulation (0.08). The probability of fecundity (i.e., of copulation potentially resulting in conception) was highest on days three and four of the MSP (0.24), and steadily decreased to probabilities below 0.10 by day fourteen. The high variability in the timing of ovulation combined with the fact that some females had long MSPs resulted in very low day-specific probabilities of fecundity. When all cycles from our study were pooled together, high fecundity was not concentrated around specific days of the MSP. This resulted in a high proportion of cycle days when the probability of fecundity was low, but above zero, for the females.

## Discussion

This study provides detailed information about reproductive hormone levels–urinary E1 and Pd–the timing of ovulation, and associated swelling patterns in wild female bonobos. Assessment of sexual swelling patterns revealed that the duration of females’ MSPs was highly variable. Furthermore, there was high variability in the timing of ovulation relative to the onset of the MSP, with ovulation occurring outside of the MSP in almost one third of the ovulatory cycles. Since ovulation did not occur in an additional eight swelling cycles, ovulation occurred during the MSP in only 52.9 % of the analysed cycles. These findings resulted in a very low probability of ovulation occurring on any particular day of a female’s MSP, suggesting that the sexual swellings of wild bonobos are a poor indicator of the timing of ovulation within cycles, and do not always signal fecundity across cycles.

Female dominance rank was not found to have a significant effect on the ISI duration, the MSP duration, the occurrence of ovulation, or the timing of ovulation. Some studies suggested that lower-ranking females may experience suppressed reproductive functioning due to harassment from higher-ranking individuals [92]. If social subordination inhibits the oestrogen-induced surge in luteinising hormone, this can result in anovulatory cycles or premature termination of menstrual cycles [101]. However, aggressive interactions between females are relatively infrequent in bonobos [63, 102]. Dominant females show tolerance toward lower-ranking females, and conflicts are often resolved in non-agonistic ways without overt aggression. Additionally, since several females shared the same dominance rank in our study (Table 1), there was not a steep dominance hierarchy among the females. Access to food resources and the nutritional status of female primates can influence their reproductive hormone levels [103, 104], fecundity (e.g., [105]), and the size and duration of sexual swellings [30]. However, differences in energetic condition and access to resources may be mitigated by the tolerant nature of bonobos [106], and by reduced levels of feeding competition compared to other species [107] (but see [108]). The relationship between feeding ecology and female rank in bonobos remains an area for future investigation.

### Duration of the interswelling interval (ISI) and the interovulatory interval (IOI)

The mean duration of ovarian cycles for females in this community, determined from the ISI and the IOI, is within the range previously reported for the intermenstrual interval of bonobos in captivity ($\overline{X}$ = 33.8–49 days) [52, 57, 58, 109–111] and in the wild ($\overline{X}$ = 42 days) [59]. Differences in cycle duration are likely due to variability in follicular phase duration, as has been found in other studies [52, 112], and as can be inferred from the relatively constant duration of the luteal phase found in our study. Several factors including lactation [52, 113], dominance rank [91], and age [112, 114] have been found to influence duration of the follicular phase. More specifically, mean cycle duration was significantly longer for low-ranking gelada baboons [92] and for lactating captive bonobos [53]. Although there was considerable interindividual and intraindividual variation in our study, dominance rank and reproductive state did not have a significant effect on the ISI duration. The observed variation in the ISI duration merits further investigation.

There was less variability in the IOI compared to the ISI. This suggests that ovulation may be relatively stable temporally, while the onset of the MSP, and swelling patterns in general, vary according to external influences (e.g., social or ecological factors) or due to physiological factors. For example, the consumption of plant steroids can influence endogenous hormone levels [115] and resulted in the suppression of sexual swellings in wild baboons [116]. In other studies, elevated cortisol levels were linked to extended cycle lengths [117] and reproductive suppression [118, 119]. If the IOI remains constant across multiple cycles, variability in the timing of ovulation relative to the MSP might be caused by variability in the onset and duration of the MSP. Accurate assessment of this phenomenon would require the collection of multiple consecutive cycles from the same female.

### High variability in the duration of the maximum swelling phase (MSP)

The mean MSP duration ($\overline{X}$ ± SD = 10.6 ± 6.8 days) was shorter in the Luikotale community compared to wild bonobos at Wamba ($\overline{X}$ = 14.6 days [59]) and bonobos in captivity ($\overline{X}$ = 16.0 ± 6.8 days [53]; $\overline{X}$ = 11.5 days [52]). This could be due in part to our conservative estimate of the MSP duration when there was a sample gap of one day at the onset or end of the MSP. Additionally, studies show that captive primates who are well-nourished and have improved nutritional condition, compared to their wild counterparts, have enhanced reproductive and hormonal functioning [104, 120]. A shorter mean duration of the MSP could be caused by fluctuations in food availability and energetic costs. These fluctuations are likely higher for bonobos in the wild, compared to bonobos in captivity or at research sites during periods when they were provisioned with food [59].

Although there was considerable variation in the MSP duration of the Luikotale bonobos (range: 1–31 days), this finding corroborates other studies of captive bonobos, MSP range: 4–26 days [52] and 3–30 days [53], as well as a study of wild bonobos which reported a MSP range of 3–22 days [59]. The variability in MSP duration for bonobos exceeds the variability found in populations of wild chimpanzees at Taï National Park, Côte d’Ivoire ($\overline{X}$ = 10.9 days, range: 6–18 days, [16]), at Mahale ($\overline{X}$ = 11.3 days, range: 5.0–14.0 days, [121]), and at Gombe ($\overline{X}$ = 9.6 days, range: 7–17 days, [122]).

Consistent with Reichert et al. [53], neither female parity nor reproductive state had a significant effect on the MSP duration. This finding differs from several other species of nonhuman primates, including some populations of chimpanzees, where nulliparous females often have longer MSPs compared to parous females [95, 123]. At Luikotale, females’ MSPs generally increased in duration as time since parturition increased; however, the number of days since parturition did not have a statistically significant effect on MSP duration. Shorter MSPs following parturition could be caused by the energetic demands of lactation [124] and an associated decrease in ovarian steroid production [99]. However, some of our females had swelling cycles with prolonged MSPs while lactating. Contrary to similar studies on other species of nonhuman primates [16, 38], we found that MSP duration did not vary significantly between individuals. This may be due to the large intraindividual variability in MSP duration across different cycles of some females. High intraindividual and interindividual variability in MSP duration contribute to bonobo sexual swellings being less precise signals of ovulation, and render it difficult for male bonobos to predict the timing of ovulation.

### Anovulatory swelling cycles in wild bonobos

There are only a few cases of anovulatory cycles in nonhuman primates reported in the literature [52, 125, 126]. During our study, we observed eight swelling cycles with no indication that ovulation occurred, yet females displayed sexual swellings with MSPs that resembled patterns of normal, ovulatory cycles (see Fig. 6 and Additional file 3: Table S8). Generally, changes in the size of female sexual swellings are regulated by oestradiol and progesterone [41, 43]. In the absence of a pronounced pre-ovulatory rise in oestrogen, swelling tumescence may result from changes in oestrogen and progestin receptor concentration in the sexual skin [127], in combination with small fluctuations in oestrogen and progesterone levels. In comparison to other species which do not display sexual swellings decoupled from ovulatory cycles, the receptor sensitivity and density of bonobo sexual swellings may be different, or may fluctuate in a different way in relation to hormone excretion. Furthermore, it is possible that oestrogen is metabolised at or near oestrogen receptors in target tissues, e.g., in female sexual swellings, but that this metabolism is not reflected in urinary measurements of oestrogen metabolites [128].

In addition to bonobo sexual swellings being a relatively weak intracycle signal of the fecund phase, the presence of tumescent sexual swellings when females are not ovulating exemplifies the low intercycle reliability of this signal across the interbirth interval. As reported in other studies [62, 65], females displayed maximally tumescent sexual swellings during periods of gestation. They also displayed MSPs as early as three months (this study) to eight months [62] following parturition, during which time ovulation is unlikely. These findings parallel studies that found that female primates displayed situation-dependent sexual swellings during certain events, e.g., encounters with strange males or group takeovers by a new male [47, 129]. Other researchers have proposed that sexual swellings may function as a social passport during intergroup transfer and immigration to enhance social integration of females [130–132]. Since young, nulliparous females often do not give birth for several years after immigration, it is possible that they are displaying sexual swelling cycles during this time without ovulating, or that the quality of these cycles is not sufficient for conception. Females may display maximally tumescent swellings which are decoupled from ovulation during these situations to appear receptive and attractive to males [133]. If tumescent sexual swellings are perceived to signal female fecundity and the ability to conceive, they could facilitate social interactions and integration with both males and females, e.g., mothers of potential male mating partners. Furthermore, sexual swellings decoupled from ovulation may be used in a strategic way by females with young, dependent offspring, enabling females to appear sexually attractive and receptive to males without incurring the risk of conceiving. Given these potential social functions of sexual swellings, it is possible that sexual swellings during periods of low or zero fecundity may not have been selected against, as they might facilitate female immigration and social interactions.

### High variability in the timing of ovulation results in low predictability of ovulation and fecundity

Previous studies spanning several species of primates (e.g., *Macaca nigra, Papio cynocephalus*: reviewed in [28]; *Pan troglodytes spp*: [16, 134, 135]) reported some variation in the temporal relation between ovulation and sexual swellings; however, ovulation almost always occurred during the second half of the MSP. A study on captive bonobos found greater variability in the timing of ovulation relative to patterns of sexual swelling; however, the variability was limited to the second half of the MSP and post-detumescence [53]. Our results from wild bonobos show even more variability in the timing of ovulation, with ovulation occurring before, after, or on almost any day of the MSP. Given that ovulation occurred during the MSP in only 52.9 % of the analysed swelling cycles, female bonobos appear to be an extreme example of variability in the timing of ovulation relative to the sexual swelling signal. If we conceptualise the signal reliability of sexual swellings as a continuum, species with swellings that reliably or accurately signal the timing of ovulation would be distributed at one end of the continuum. Our findings suggest that wild bonobos occupy a position towards the opposite end of the continuum, where sexual swellings indicate ovulation with much less reliability and accuracy than in other species.

Consequently, the day-specific probabilities of ovulation and fecundity for female bonobos were very low, especially when compared to the same probabilities in female Western chimpanzees at Taï [16] (Fig. 7b). At its highest calculated value (0.24), the probability of a female bonobo being fecund, i.e., able to conceive, was two and a half times lower than in other species of primates, e.g., *Pan troglodytes verus*: 0.64 [16] and *Hylobates lar*: 0.73 [38]. The low predictability of ovulation in wild bonobos may hinder male mate guarding of females, especially when several females display MSPs simultaneously [133, 136]. Overlap in females’ MSPs often occurs in bonobos due to the lengthy duration of the MSP within a cycle and the high number of swelling cycles within interbirth intervals of females. Reproductive synchrony and temporal overlap in female receptivity or oestrous have been found to inhibit male monopolisation potential in other species [137, 138], and may affect male mating strategies in bonobos as well.

### Broader Implications

In species where the timing of ovulation within a cycle can be more accurately predicted, males may be able to mate guard and monopolise fecund females during days when they are able to conceive. In a number of primate species, mate guarding is often used as a form of indirect sexual coercion by males [139] to constrain with whom a female can mate, and thereby ensure that the mate-guarding male sires a female’s offspring. In communities of chimpanzees where ovulation usually occurs near the end of the MSP and is thus relatively predictable, some studies found high frequencies of male-male mate competition and corresponding high levels of testosterone in high-ranking males when females exhibited MSPs (e.g., [140] but see [141]).

Given the low predictability of ovulation and fecundity in wild bonobos based on sexual swelling patterns alone, males may have to attend to other cues and signals to correctly pinpoint a female’s fecund phase and time their mating efforts effectively. Chemosignals [142–144], behavioural cues [125], and vocal cues [145, 146] may play ancillary roles in signalling female fecundity in bonobos, as has been found in other species. If males are using other signals and focussing their mating efforts accordingly, this may offer one explanation as to why a high proportion of copulations are observed outside the MSP in bonobos, compared to other species [58, 60, 147]. Our results show that ovulation occurred outside the MSP in over 30 % of the ovulatory cycles we analysed. If males are able to detect ovulation by behavioural, olfactory, or other signals, this could cause males to solicit females outside the MSP and result in a weaker correlation between male solicitations and the MSP.

When ovulation is not tightly linked to the MSP, and if males are not able to discern and predict the window of fecundity in a female’s cycle using other signals, then it is more difficult and costly for a male to monopolise a female over a long period of time [148]. The costs associated with mate guarding a female throughout an extended MSP may outweigh the benefits [30]. Consequently, the high degree of uncertainty in bonobo sexual swellings may constrain or eliminate mate guarding by males. This might have resulted in male bonobos adopting alternative mating strategies to increase their mating success, e.g., deferring to females in feeding contexts, grooming females, or being perpetually willing to copulate with females. Accordingly, alternative mating strategies may lead to males investing more into affiliative relationships with females [149] rather than competing with other males for mating opportunities. In support of this theory, a recent study in this community found that high-ranking male bonobos with the highest mating success did not have elevated levels of testosterone during periods of mate competition [68]. In contrast to many communities of chimpanzees, these results suggest that male bonobos are not using intrasexual aggression to compete over potentially fecund females.

By displaying tumescent sexual swellings during extended periods of time, female bonobos may prolong the period during which they are attractive to males, and thereby increase their ability to confuse paternity [150]. Furthermore, if high costs associated with mate guarding and aggressing females lead to a decrease in these behaviours by males, females may be less constrained in expressing female mate choice compared to other species [151]. If female bonobos can manipulate male mating strategies or benefit from having sexual swellings that are less reliable and of longer duration, then one might ask why female chimpanzees do not have sexual swellings similar to those of female bonobos? The cost-of-sexual-attraction hypothesis may offer one explanation [107]. This hypothesis posits that the intensity of female sexual attractiveness, exhibited via sexual swellings, is primarily driven by levels of within-group scramble competition. If ranging in large groups and maintaining sexual swellings is costly for females due to low food availability, insufficient food between fruit trees, e.g., terrestrial herbaceous vegetation, or anthropogenic disturbances, then females might intensify their sexual attractiveness during shorter periods of time [152]. This, in turn, could lead to elevated levels of sexual coercion by males [153]. On the other hand, a low cost of grouping could lead to a lower intensity of sexual attractiveness, i.e., longer MSPs, and could increase the number of cycles per conception, resulting in a lower level of sexual coercion and more social benefits for females. Although this hypothesis seems plausible, ecological data to support it are still lacking. Empirical data pertaining to the quality and quantity of available food resources and the cost of grouping in wild bonobos will contribute to understanding how sexual swellings affect ranging and behaviour patterns in bonobos versus chimpanzees.

The extent to which male bonobos attend to female sexual swellings and use these signals to time their mating efforts and strategies remain to be investigated. Further study will assess whether there is evidence of female mate choice in wild bonobos, whether females modify their mating strategies across the ovarian cycle, and to what extent sexual swellings serve other social functions in this species.

## Conclusions

We found a weak temporal relationship between sexual swellings and the timing of ovulation in wild female bonobos, which resulted in a very low day-specific probability of ovulation and fecundity during a female’s MSP. Bonobo sexual swellings appear to send mixed messages to males, as they do not always signal fecundity or imminent ovulation. Hence they are only probabilistic signals, rather than reliable indicators of ovulation. High variability in the relation between this sexual signal and the timing of ovulation may make it difficult for males to accurately time their mating efforts, if they use sexual swellings alone to assess female fecundity. By inference, it is likely that other sources of information, e.g., behavioural cues from females, trigger reproductive investment of males. If the temporal inflation and variability of sexual swellings in relation to ovulation constrains mate-guarding efforts by male bonobos, this could enable females to express mate choice without being constrained by males. Such a scenario would support models that emphasise differences in patterns of sexual conflict between bonobos and chimpanzees [1, 154].

## Abbreviations

Cr, creatinine; E1, Oestrone; GLMM, generalised linear mixed model; IOI, interovulatory interval; ISI, interswelling interval; LMM, linear mixed model; MSP, maximum swelling phase; Pd, pregnanediol.

## Additional files

Additional file 1: Further details of hormone extraction and measurement. (PDF 334 kb) Additional file 2: Summary of results from the LMMs and GLMMs: **Table S1.** the ISI duration GLMM; **Tables S2–S4.** the three MSP duration LMMs; **Table S5.** the female rank and occurrence of ovulation GLMM; **Table S6.** the female rank and timing of ovulation GLMM. (PDF 331 kb) Additional file 3: Supplemental information: **Table S7.** Details of ovulatory cycles; **Table S8.** Details of anovulatory cycles. (PDF 209 kb)
