# Supplementary material for: Mixed messages: wild female bonobos show high variability in the timing of ovulation in relation to sexual swelling patterns
Source: BMC Evol Biol. 2016 Jun 30;16:140. doi: 10.1186/s12862-016-0691-3 (PMC4928307; doi:10.1186/s12862-016-0691-3)
Supplement: Additional file 1: — Further details of hormone extraction and measurement. (PDF 334 kb) [file 12862_2016_691_MOESM1_ESM.pdf]

Additional file 1 for “Mixed messages: wild female bonobos show high variability in the timing of ovulation in relation to sexual swelling patterns”

Authors: P. H. Douglas, G. Hohmann, R. Murtagh, R. Thiessen-Bock, T. Deschner

Further details of hormone extraction and measurement

The following method of steroid hormone extraction is adopted from Hauser et al. [1].

Hormone extraction

Following hydrolysis, we performed solid phase extraction (SPE) on polymer-based reversed phase cartridges (Chromabond® HR-X, 30mg, Macherey-Nagel, Düren, Germany). Steroids were reconstituted in phosphate buffer; subsequently, free steroids were separated from conjugated steroids by liquid-liquid extraction (LLE) with ether.

Solvolysis was performed on the sulfate conjugates from the aqueous phase using a mixture of ethyl acetate and sulfuric acid. We performed a second LLE to further separate free steroids. Both organic phases containing the free steroids were combined and measured by LC-MS/MS.

Hormone measurement

Pregnanediol was measured in the positive mode (ESI+); oestrone (E1) was better quantified in the negative mode (ESI-) due to peak interference in ESI+. Analyte separation was achieved at 30°C on a reverse phase C18 column (Gemini C18, 150mm x 2mm, 3µm, Phenomenex, Torrance, CA). Intra- and inter-CV, calculated from replicate measurements of quality controls, were 8.09% and 15.33% for Pd.

For ESI+ analysis, mobile phases A and B were composed of acetonitrile/water solutions, 5/95 (v/v) and 95/5 (v/v), respectively, both containing 0.1% formic acid. A gradient elution at 0.2ml/min was 30% B (0–2 min), linear increase to 70% B (2–20 min), 90% B (21–24 min), and 30% B (24–34 min). For ESI- analysis, mobile phases A and B were composed of acetonitrile/water solutions, 5/95 (v/v) and 95/5 (v/v) respectively, both containing 0.1% ammonium hydroxide. A gradient elution at 0.2ml/min was 30% B (0–1 min), linear increase to 90% B (1–10 min), and 30% B (13–22 min). Intra- and inter-CV, calculated from replicate measurements of quality controls, were 6.21% and 8.54% for E1.

Desolvation and cone gases were nitrogen (NGM-11 nitrogen generator, CMC Instruments, Eschborn, Germany), with flow rates of 900L/h and 250 L/h, respectively. Argon was used as the collision gas. Source and desolvation temperatures were 100 and 450°C, respectively. Capillary voltage was applied at 3.8V. Cone voltage and collision energy were set individually for each compound. The hormones of interest were detected using multiple reaction monitoring (MRM) of the two most abundant product ions per analyte.

Reference

1. Hauser B, Deschner T, Boesch C. Development of a liquid chromatography–tandem mass spectrometry method for the determination of 23 endogenous steroids in small quantities of primate urine. J of Chromatogr. B. 2008; 862:100–112.
