# Supplementary material for: Mixed messages: wild female bonobos show high variability in the timing of ovulation in relation to sexual swelling patterns
Source: BMC Evol Biol. 2016 Jun 30;16:140. doi: 10.1186/s12862-016-0691-3 (PMC4928307; doi:10.1186/s12862-016-0691-3)
Supplement: Additional file 2: — Summary of results from the LMMs and GLMMs: Table S1. the ISI duration GLMM; Tables S2–S4. the three MSP duration LMMs; Table S5. the female rank and occurrence of ovulation GLMM; Table S6. the female rank and timing of ovulation GLMM. (PDF 331 kb) [file 12862_2016_691_MOESM2_ESM.pdf]

Additional file 2 for "Mixed messages: wild female bonobos show high variability in the timing of ovulation in relation to sexual swelling patterns"

Authors: P. H. Douglas, G. Hohmann, R. Murtagh, R. Thiessen-Bock, T. Deschner

Table S1. Results from the model of Interswelling Interval (ISI) duration. (A GLMM with female ID as a random effect.)

Term	Estimate	SE	χ^2	Df	P	Lower CL	Upper CL
intercept	3.698	0.079	(4)	(4)	(4)	3.302	4.064
parity - nulliparous(1)	-0.031	0.435	0.373(5)	2	0.830(5)	-0.839	0.877
parity - primiparous(1)	0.075	0.283				-0.444	0.644
reproductive state(2)	0.031	0.092	0.115	1	0.735	-0.145	0.231
female rank(3)	-0.046	0.158	0.086	1	0.769	-0.389	0.235

(1) dummy coded with multiparous being the reference level

(2) 0 = cycling; 1 = early lactation (0–24 months since parturition)

(3) z-transformed to a mean of zero and a standard deviation (SD) of one (Mean and SD for the original variable were 3.270 and 2.256, respectively.)

(4) not indicated because of not having a meaningful interpretation

(5) the test results shown here indicate the overall significance of parity

Table S2. Results from the first model of MSP duration. (A LMM with female ID as a random effect, and random slopes of days since parturition as well as the two season effects within female ID.)

Term	Estimate	SE	χ^2	Df	P	Lower CL	Upper CL
intercept	2.728	0.681	(5)	(5)	(5)	1.332	4.205
parity(1)	-0.934	0.693	1.341	1	0.247	-2.307	0.423
days since parturition	0.034	0.017	4.215	1	0.040	-0.003	0.070
reproductive state(2)	-0.630	0.400	2.398	1	0.122	-1.448	0.153
female rank(3)	0.277	0.312	0.643	1	0.423	-0.344	0.905
sin(season)(4)	0.326	0.258	1.978	2	0.372	-0.189	0.808
cos(season)(4)	-0.109	0.210				-0.520	0.340

(1) 0 = multiparous; 1 = primiparous

(2) 0 = not cycling; 1 = cycling

(3) z-transformed to a mean of zero and a standard deviation (SD) of one (Mean and SD for the original variable were 2.415 and 1.622, respectively.)

(4) the test results shown in the row sin(season) indicate the overall significance of season, obtained from comparing the full model with a reduced model lacking the two effects

(5) not indicated because of not having a meaningful interpretation

Table S3. Results from the second model of MSP duration. (A LMM with female ID as a random effect, and random slopes of days since parturition as well as the two season effects within female ID.)

Term	Estimate	SE	χ^2	Df	P	Lower CL	Upper CL
intercept	2.436	0.688	[4]	[4]	[4]	0.924	3.932
days since parturition	0.032	0.019	2.879	1	0.090	-0.007	0.072
reproductive state[1]	-0.574	0.392	1.970	1	0.160	-1.397	0.321
female rank[2]	-0.076	0.196	0.149	1	0.699	-0.515	0.324
sin(season)[3]	0.318	0.254	2.131	2	0.345	-0.195	0.834
cos(season)[3]	-0.135	0.209				-0.578	0.299

(1) 0 = not cycling; 1 = cycling

(2) z-transformed to a mean of zero and a standard deviation (SD) of one (Mean and SD for the original variable were 2.415 and 1.622, respectively.)

(3) the test results shown in the row sin(season) indicate the overall significance of season, obtained from comparing the full model with a reduced model lacking the two effects

(4) not indicated because of not having a meaningful interpretation

Table S4. Results from the third model of MSP duration. (A LMM with female ID as a random effect, and random slopes of days since parturition as well as the two season effects within female ID.)

Term	Estimate	SE	χ^2	Df	P	Lower CL	Upper CL
intercept	2.551	0.681	[4]	[4]	[4]	1.082	3.95
parity[1]	-0.408	0.422	0.847	1	0.357	-1.288	0.376
days since parturition	0.034	0.018	3.380	1	0.066	-0.003	0.073
reproductive state[2]	-0.589	0.393	2.114	1	0.146	-1.424	0.226
sin(season)[3]	0.317	0.255	2.135	2	0.344	-0.203	0.839
cos(season)[3]	-0.139	0.207				-0.552	0.251

(1) 0 = multiparous; 1 = primiparous

(2) 0 = not cycling; 1 = cycling

(3) the test results shown in the row sin(season) indicate the overall significance of season, obtained from comparing the full model with a reduced model lacking the two effects

(4) not indicated because of not having a meaningful interpretation

Table S5. Results from the model of occurrence of ovulation. (A GLMM with female ID as a random effect.)

Term	Estimate	SE	χ^2	Df	P	Lower CL	Upper CL
intercept	1.448	0.661	(3)	(3)	(3)	0.488	7.141
female rank(1)	0.173	0.529	0.110	1	0.740	-0.937	1.958
reproductive state(2)	-0.494	0.950	0.272	1	0.602	-3.535	14.709

(1) z-transformed to a mean of zero and a standard deviation (SD) of one (Mean and SD for the original variable were 2.971 and 1.696, respectively.)

(2) 0 = cycling; 1 = early lactation

(3) not indicated because of not having a meaningful interpretation

Table S6. Results from the model of timing of ovulation. (A GLMM with female ID as a random effect.)

Term	Estimate	SE	χ^2	Df	P	Lower CL	Upper CL
intercept	1.348	0.586	(3)	(3)	(3)	0.513	8.975
female rank(1)	0.607	0.524	1.482	1	0.223	-0.391	4.304
reproductive state(2)	-1.447	0.983	2.211	1	0.137	-17.410	0.787

(1) z-transformed to a mean of zero and a standard deviation (SD) of one (Mean and SD for the original variable were 3.038 and 1.886, respectively.)

(2) 0 = cycling; 1 = early lactation

(3) not indicated because of not having a meaningful interpretation
