# Supplementary material for: Mixed messages: wild female bonobos show high variability in the timing of ovulation in relation to sexual swelling patterns
Source: BMC Evol Biol. 2016 Jun 30;16:140. doi: 10.1186/s12862-016-0691-3 (PMC4928307; doi:10.1186/s12862-016-0691-3)
Supplement: Additional file 3: — Supplemental information: Table S7. Details of ovulatory cycles; Table S8. Details of anovulatory cycles. (PDF 209 kb) [file 12862_2016_691_MOESM3_ESM.pdf]

Additional file 3 for "Mixed messages: wild female bonobos show high variability in the timing of ovulation in relation to sexual swelling patterns"

Authors: P. H. Douglas, G. Hohmann, R. Murtagh, R. Thiessen-Bock, T. Deschner

Table S7. Details of ovulatory cycles. Cycles are listed alphabetically by female identity then chronologically, to correspond with Fig. 5.

Female	Cycle	Rank	Reproductive State	Months since parturition	Timing of ovulation relative to the MSP		
					Before	During	After
Luna	(I)	5	Cycling	[(1)]			After
Luna	(II)	5	Cycling	[(1)]		During	
Luna	(III)	5	Cycling	[(1)]		During	
Luna	(IV)	5	Cycling	[(1)]		During	
Martha	(I)	1	Cycling	> 60		During	
Martha	(II)	1	Cycling	> 60		During	
Martha	(III)	1	Cycling	> 60	Before		
Martha	(IV)	1	Cycling	> 60		During	
Olga	(I)	2	Early lactation	20	Before		
Olga	(III)	2	Cycling	31			After
Olga	(IV)	2	Cycling	33		During	
Olga	(V)	2	Cycling	34		During	
Paula	(I)	1	Early lactation	6		During	
Paula	(II)	1	Early lactation	9			After
Paula	(III)	1	Early lactation	19			After
Rio	(I)	2	Cycling	> 60		During	
Rio	(II)	2	Cycling	> 60		During	
Susi	(I)	6	Early lactation	21		During	
Susi	(II)	6	Cycling	35		During	
Susi	(III)	6	Cycling	44		During	
Susi	(IV)	6	Cycling	46		During	
Uma	(II)	3	Cycling	41		During	
Uma	(IV)	3	Cycling	43		During	
Wilma	(II)	4	Early lactation	16		During	
Wilma	(IV)	4	Cycling	34			After
Zoe	(I)	2	Early lactation	8			After

[(1)] this female was nulliparous when the cycle was collected

Table S8. Details of anovulatory cycles. Cycles are listed alphabetically by female identity then chronologically, to correspond with Fig. 6.

Female	Cycle	Rank	Reproductive State	Months since parturition
Olga	(II)[(1)]	2	Early lactation	22
Rio	(IV)	2	Early lactation	11
Uma	(I)	3	Cycling	31
Uma	(III)	3	Cycling	42
Wilma	(I)	4	Early lactation	14
Wilma	(III)	4	Cycling	26
Zoe	(II)	2	Cycling	26
Zoe	(III)	2	Cycling	33

[(1)] ovulation was detected in a swelling cycle prior to this cycle, during the period of early lactation (see Table S7)
